# Supplementary material for: Simulating DNA Chip Design Using All-Electronic Graphene-Based Substrates
Source: Molecules. 2019 Mar 8;24(5):951. doi: 10.3390/molecules24050951 (PMC6429485; doi:10.3390/molecules24050951)
Supplement: Supplementary file 1 [file molecules-24-00951-s001.pdf]

Supplementary Materials: Simulating DNA chip design using all-electronic graphene-based substrates

Ernane de Freitas Martins [1,2,†], Gustavo Troiano Feliciano [3], Ralph Hendrik Scheicher [2] and Alexandre Reily Rocha [1,*]

1. Convergence analysis

A convergence analysis of the electronic transport calculation is shown in Figures **??** and **??** for the vertical and horizontal setups, respectively. This analysis is performed by taking the the evolution of the average over the specified number of electronic transport calculations for a specific energy ($E \approx 0.0\ eV$). As can be observed, both transmission and standard deviation (SD) are converged for 50 frames.

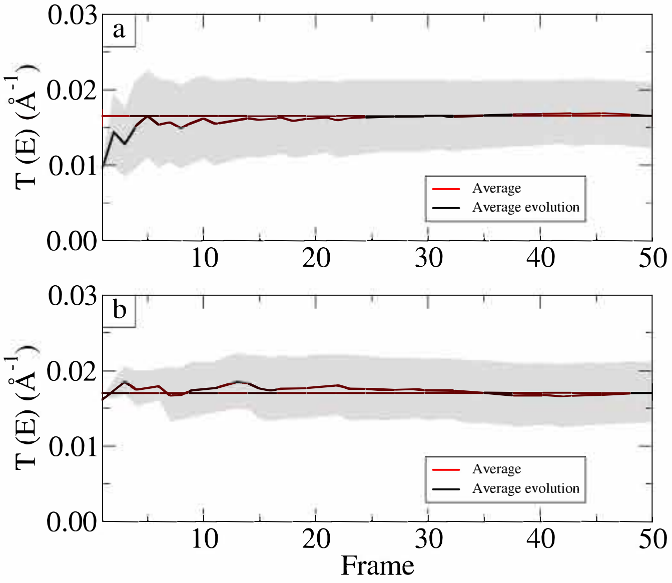

Figure S1. Evolution of the average and SD for vertical (a) ssDNA and (b) dsDNA systems. The gray shaded region comprises the addition and subtraction of 1 SD in each calculated point. Chosen energy $E \approx 0.0\ eV$.

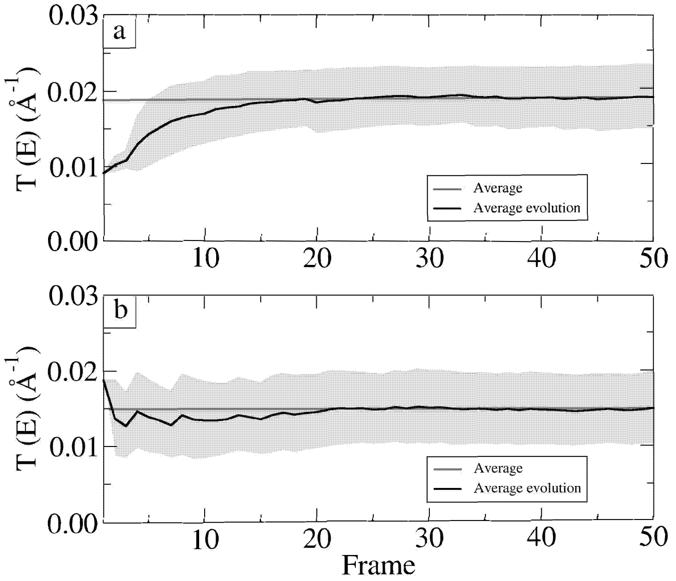

Figure S2. Evolution of the average and SD for horizontal (a) ssDNA and (b) dsDNA systems. The gray region comprises the addition and subtraction of 1 SD in each calculated point. Chosen energy $E \approx 0.0\, eV$.

Figures ?? and ?? show the average electronic transport calculation for ssDNA and dsDNA in the vertical and horizontal setups, respectively. The gray area in the figures comprises the addition and subtraction of one SD.

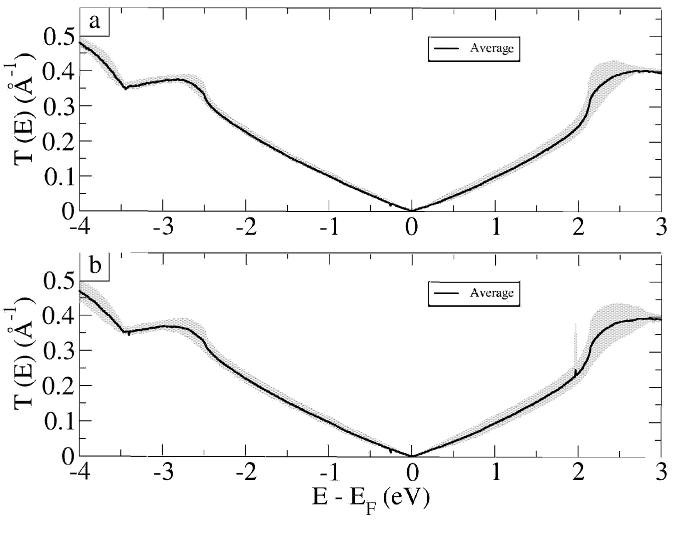

Figure S3. Average electronic transport calculation for (a) ssDNA and (b) dsDNA in the vertical setup. The gray region comprises the addition and subtraction of 1 SD in each calculated point.

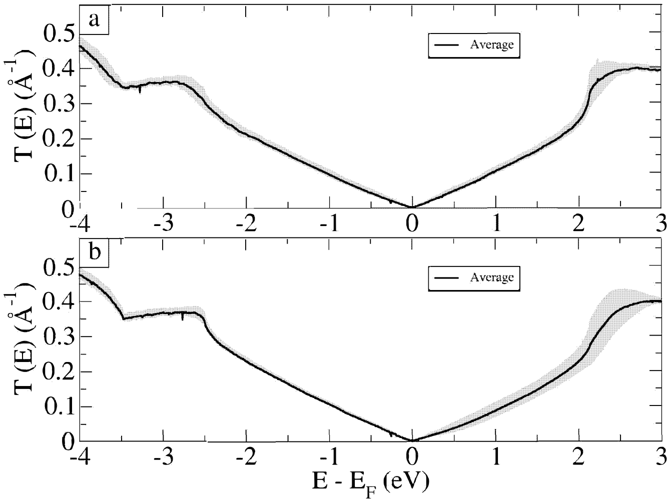

Figure S4. Average electronic transport calculation for (a) ssDNA and (b) dsDNA in the horizontal setup. The gray region comprises the addition and subtraction of 1 SD in each calculated point.

2. Structural analysis

The root mean square deviation (RMSD) for ssDNA and dsDNA in both vertical and horizontal setups is shown in Figure ??. This graph is constructed by interpolating the data from the analysis to have a clean view. As can be observed in both Figures ??a and ??b, the deviation from a reference structure for vertical setup is always larger than the deviation for horizontal setup. This means that the structural fluctuation in the vertical setup is larger than the case of horizontal setup. In summary, the results presented in Figure ?? show why the device's selectivity in the vertical setup present more noize compared with the horizontal setup.

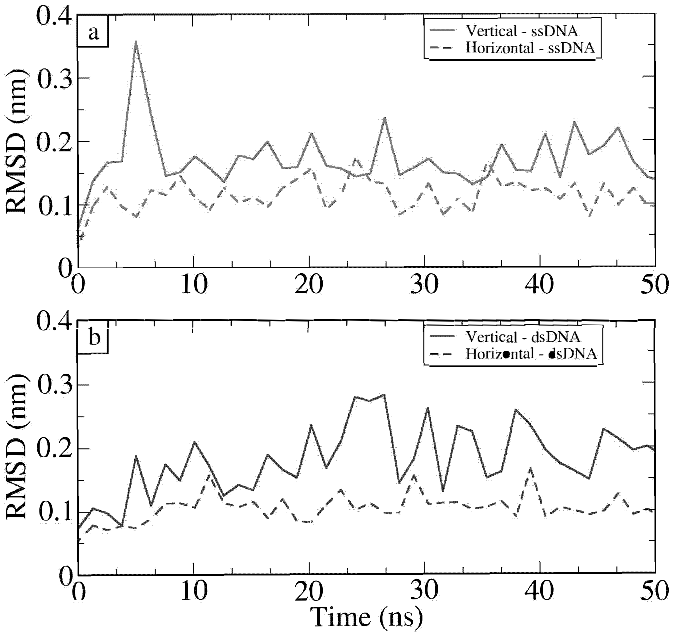

Figure S5. RMSD for 50 ns of the simulation for (a) ssDNA and (b) dsDNA systems in both vertical and horizontal setups.
